# An Investigation of the Work Hardening Behavior in Interrupted Cutting Inconel 718 under Cryogenic Conditions

**DOI:** 10.3390/ma13092202

**Published:** 2020-05-11

**Authors:** Xing Dai, Kejia Zhuang, Donglin Pu, Weiwei Zhang, Han Ding

**Affiliations:** 1 State Key Laboratory of Digital Manufacturing Equipment and Technology, Huazhong University of Science and Technology, Wuhan 430074, China; daixing_hust@163.com (X.D.); cheungxm@gmail.com (H.D.); 2 Hubei Digital Manufacturing Key Laboratory, School of Mechanical and Electronic Engineering, Wuhan University of Technology, Wuhan 430070, China; zwwvivid@whut.edu.cn; 3 Wuxi Research Institute of Huazhong University of Science and Technology, Wuxi 214100, China; pudl@hust-wuxi.com

**Keywords:** cryogenic machining, work hardening layer, turning, Inconel 718

## Abstract

The severe work hardening phenomenon generated in the machining of Inconel 718 is harmful to continue cutting processes, while being good for the component’s service performance. This paper investigates the performance of cryogenic assisted machining used in the cutting processes, which can reduce the waste of fluids. The influence of dry and cryogenic machining conditions with different cutting speeds on the work hardening layer is investigated based on the interrupted cutting of Inconel 718. Cutting temperature distribution obtained from simulations under different conditions is used to discuss the potential mechanism of work hardening. Then, the depth of work hardening and degree of work hardening (DWH) are investigated to analyze the surface deformation behavior, which strengthens the machined surface during metal cutting processes. From the cutting experiments, the depth of the work hardening layer can reach more than 60 μm under the given cutting conditions. In addition, a deeper zone can be obtained by the cooling of liquid nitrogen, which may potentially enhance the wear performance of the component. The results obtained from this work can be utilized to effectively control the work hardening layer beneath the surface, which can be applied to improve the service performance.

## 1. Introduction

As one of the super alloys containing a niobium age-hardening addition, Inconel 718 takes advantage of its very high strength and anti-fatigue properties [1]. This material has been widely used in the manufacture of components for liquid rockets, as well as parts for aircraft turbine engines, cryogenic tankage, etc. [2]. The mechanical properties of Inconel 718 are greatly enhanced by its austenitic face-centered cubic (FCC) crystal structure with high phase stability. One of the factors that results in difficulty in the processing of this material is the hardening effects of the two phases: gamma double prime (γ’’) precipitates; and gamma prime (γ’) precipitates [3]. The cutting temperature would rise drastically, and plastic deformation of the cutting tool eventually takes place with the low thermal conductivity during machining of Inconel 718 [4]. Thus, this material is very hard to machine compared with conventional materials. As a consequence, the high-performance machining of this kind of material is a promising research area for the public.

Inconel 718 is one of the most difficult-to-cut materials, due to its toughness, lower thermal conductivity and easy work hardening properties. Furthermore, as a kind of easy work hardening material, Inconel 718 suffers several plastic deformations during the machining process. Ultan and Ozel [5] pointed out that Inconel 718 easily forms a hardening layer, which may be caused by the thermal-mechanical loads of the cutting zone. The hardening layer beneath the machined surface can enhance the service performance of final products, while it is extremely hard for the cutting operations in sequence [6]. The formed work hardening layer should be controlled, for it may be the main incentive of the notch wear mechanism of cutting tools. Zhuang et al. [7] studied the relationship between the notch wear and work hardening layer based on experimental analysis, and noted that a control hardening layer depth exists. Different machining steps, including semi finish, finish and critical finish machining, are studied by Touazine et al. [8] to investigate the deformation zone. In their study, the deformation zone can be determined by optical images, nanoindentation and X-ray diffraction (XRD) methods. Pawade [9] studied the machine-affected zone (MAZ) beneath the surface when machining Inconel 718, and thereafter found that the microhardness of MAZ gradually approaches bulk hardness at a depth of about 300 μm below the machined surface. The work hardening layer beneath the machined surface displayed variations which are higher than the bulk hardness of the base material [10]. Dinesh et al. [11] studied the influence of machining cutting parameters on the degree of work hardening based on the experimental results. New machining parameters were selected to minimize the work hardening based on the cutting tests. Ren and Liu [12] provided a deeper explanation to understand the work hardening phenomenon during machining Inconel 718, and also noted that the hardening layer should be controlled with the influences of cutting speed and feed rate. Dynamic recrystallization and interaction are considered some of the main phenomena of plastic deformation in cutting operations, and the grain refinement effect was considered the main cause of the micro-hardness increasing [13].

The surface integrity obtained during machining of this kind of material—with assisted technologies reducing resource consumption, and thus creating less waste cutting fluid for the environment—has been studied by some scholars. In order to lower environmental impact and cost, Jose et al. [14] investigated the cutting performance of polycrystalline cubic boron nitride tools during the finish machining of Inconel 718, where they noted that the high-speed machining Inconel 718 is not acceptable in industry without any cutting fluid. Xu et al. [15] studied the surface integrity of Inconel 718 with various cooling conditions, and the result showed that the surface microhardness rate in dry milling is slightly lower than that of wet milling. Hegab and Kishawy [16,17] studied the influence of different nanofluids in minimum quantity lubrication when turning Inconel 718, and found that better surface integrity can be obtained when nanofluids are used. Musfirah et al. [18] used liquid nitrogen to cool the too-chip interface to reduce environmental problems and costs in their cutting tests, as well as to achieve better surface integrity and a longer tool life. Compared with dry machining, a better surface finish, longer tool life and lower cutting forces can be obtained during machining with cryogenic assisted technology [19]. Pusavec et al. [20] studied the cutting performance with cryogenic machining, which shows that the proposed method can be used to enhance all the major surface integrity characteristics, which can enhance the service performance of the final product. Umbrello et al. [21] noted that the cryogenic cooling method can improve the surface integrity of the machined surface, which can lead to extended product life and performance. It can be concluded that cryogenic cooling is a potential machining method for better surface integrity compared with dry conditions. The severe plastic deformation has been investigated by Yang et al. [22], who found that the surface integrity of the Co–Cr–Mo alloy can be significantly improved by the proper selection of burnishing conditions due to its refined microstructure, high hardness and favorable phase structure on the surface layer. Pereira et al. found that the small changes in microhardness occurred at a depth of 0.3 mm from beneath machine surface than in cutting with cryogenic cooling methods [23]. Zhao et al. [24] studied the effect of liquid nitrogen cooling on surface integrity in milling operations, and noted that cryogenic cooling yields higher microhardness on the surface. Zhou et al. [25] found a small difference in the mechanical state near the subsurface layer between the specimens produced with coated and uncoated cutting tools, in terms of microhardness.

It is well-accepted that the microhardness generated beneath machined surfaces has a significant influence on sequence cut and the service performance. The work hardening behavior has been investigated by numerous pieces of literature, while few are related to the effect of assisted machining methods. The insufficiency of research on work hardening behavior under cryogenic methods limits the use of the assisted machining method. Work hardening generated in cutting operations is affected by cooling methods, which should have an in-depth understanding. This paper focuses on the surface hardening behavior during the cryogenic-assisted turning of Inconel 718 with uncoated carbide cutting inserts. The effects of different cooling conditions on micro-hardness, as well as surface yield strength, are studied. The investigation illustrates the effect of cooling conditions on the yield strength base on Meyer’s law. The structure of this paper is organized as follows: Section 1 states the introduction of surface integrity obtained by cryogenic machining methods. The material and methods used in this study are illustrated in Section 2. In this section, a series of simulations with the finite element method (FEM) using AdvantEdge and cutting experiments under different cooling methods are used to illustrate the distributions of cutting temperature. The results of cutting tests under different machine conditions and discussions are given in Section 3. The depth of work hardening and prediction of yield strength beneath the machined surface are also discussed in this section. The conclusions follow this in Section 4.

## 2. Experiment and Simulation Setup

In this Section, two distinct yet complementing methods were used to study the effects of cooling methods. Cutting experiments on computer numerical control (CNC) turning center and simulations with the finite element method (FEM) are carried out separately with the same cutting conditions. Different cooling conditions (dry, cryogenic cooling the cutting zone and cryogenic precooling the workpiece) were applied to all operations to investigate their effects on the workpiece surface hardening behavior.

### 2.1. Experimental Setup

**Figure 1:**
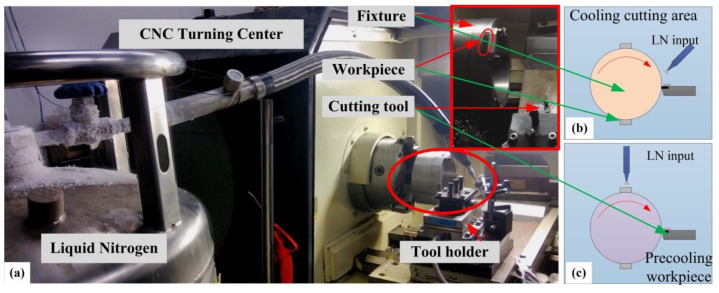
Cutting experiment setup. (**a**) Turning operation, (**b**) Cooling cutting area, (**c**) Precooling workpiece.

**Figure 2:**
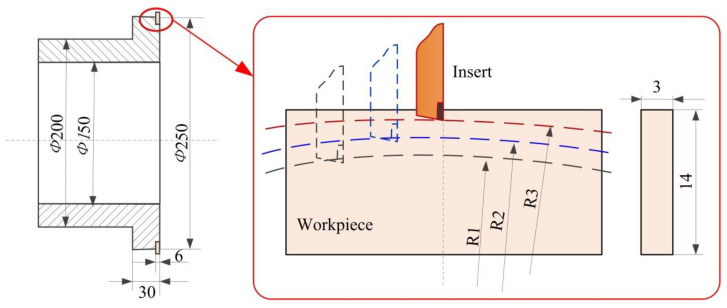
Fixture and workpiece schematic.

The CNC center made by Dalian machine tools group (Dalian, China) with a maximum speed of 3000 rpm is used to conduct the cutting experiments in this paper. The turning material used for the cutting tests is Inconel 718 with a precutting process, and Table 1 lists the mechanical composition of this material. The hardness of the bulk material (Inconel 718 by a solution heat treatment) used in this paper is about 340–360 HV_0.02_, with a density of 8.2 g/cm^3^. The experiment setup is shown in Figure 1, while the shape of the original workpiece is processed into the shapes, as shown in Figure 2. The workpiece mounted on the fixture in the cutting processes is used in the cutting tests. In the cryogenic assisted cutting process, liquid nitrogen is injected into the cooling zone, which absorbs the heat generated during machining and evaporates quickly. Figure 1b and c give the experiment’s setup of the cooling cutting area and precooling the workpiece, respectively. As shown in Figure 2, the workpieces are precut into the arc shape before the cutting experiments. Uncoated triangular carbide inserts (TCMW) are used in the cutting tests. The liquid nitrogen assisted coolant is applied to the bulk material on different locations, as shown in Table 2. Liquid nitrogen (LN2) was applied to the cutting zone or the workpiece under 1 bar pressure and 0.3 L/min flowrate. The cutting parameters and cooling method are listed in Table 2. Different cutting speeds (40 m/min, 60 m/min and 80 m/min) and assisted cooling methods (pre-cooling workpiece, cooling cutting zone) are chosen to study the influence of cutting conditions on the work hardening phenomenon.

### 2.2. Measurement and Analysis

The samples of workpieces are used for specimen preparation after the machining tests. Before sample preparation, the workpieces are cut into small samples with the size of about 3 mm × 8 mm × 8 mm, and then the smooth surface can be achieved with the polishing operation method after hot mounting. To ensure the work hardening effect on the machined surface is more accurate, the nano-indentation method was used to measure the hardness of the white layer by some researchers [25]. In this paper, the Vickers hardness tester Qness Q10A+ is used to obtain the microhardness points from the surface to bulk for the hardening layer, which is much larger than the white layer. The testing program uses a load of 20 gf, and the full load maintains for 6 s after loading.

**Figure 3:**
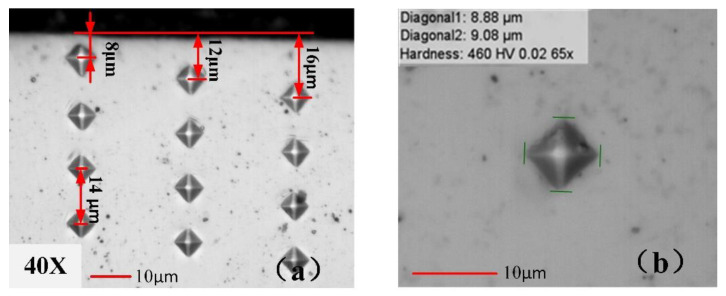
Sample of microhardness measurement (Dry cutting condition). (**a**) Test with several columns, (**b**) sample of indentation.

The sample micro indentation images are illustrated in Figure 3a, while several column test points on the cross-sections of sub-surface are measured in the testing program. Figure 3b gives samples of the identification, where the diagonal length of indentation left in the sample is about 9.0 μm, with a hardness of material of about 460 HV_0.02_. It can be seen from Figure 3a that the indentation marks near the machined surface are slightly larger than those far from the machined surface. As shown in Figure 3a, the obvious limitation of microhardness test is the size of indenter. In order to obtain more indentation within 20 μm beneath the surface, 20 gf is used in the measurement program. The micro hardness of the material near the machined surface is lower than the material far from the machined surface for the surface softening occurring near the machined surface under dry cutting conditions. The microhardness measurement is performed into a depth of 0.5 mm beneath the machined surface, according to the previous research [5]. The hardness of the bulk material is measured at a depth of about 4 mm from machined surface to ensure consistency of the testing results. The measured curves of the microhardness profile with the bulk hardness of the workpiece are used to estimate the depth of the work hardened layer.

### 2.3. Simulations with FEM

**Figure 4:**
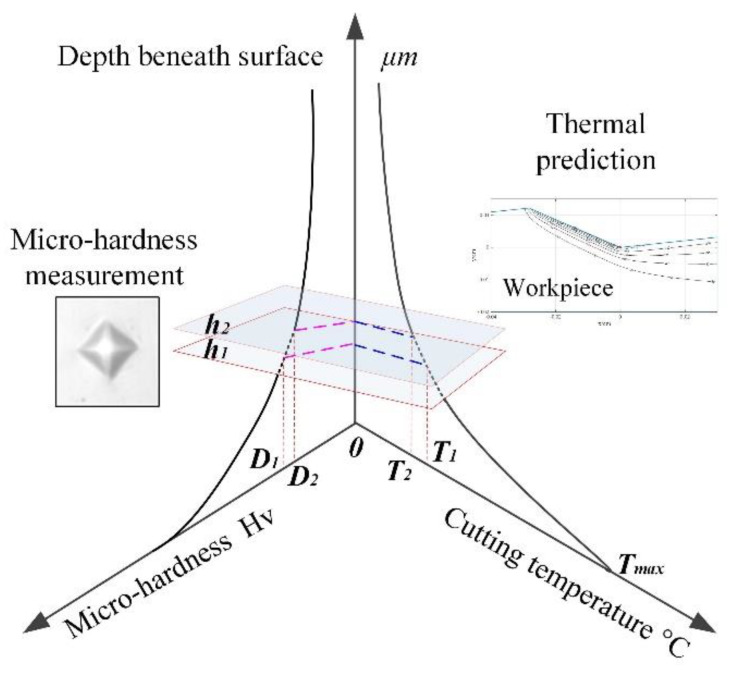
Relationship between cutting temperature and micro-hardness.

Work hardening behavior is a complex phenomenon in metal cutting processes that is affected by many factors (i.e., cutting force, cutting temperature, severe plastic strain, etc.). The high cutting temperature generated beneath the machined surface indicates that severe deformations in the crystalline structure may lead to strain strengthening in cutting operations. In addition, the cutting temperature generated in the first and third deformation zone may result in a lower scale deformation of the material, which can increase the workpiece hardness [26]. It can be inferred that the phase transformation generated beneath machined surface driven by the cutting heat, as well as the severe plastic deformation, is one of the causes of the change in micro-hardness. The machined surface achieves the highest temperature, and then decreases from the surface to depth of work hardening, as shown in Figure 4 (*h_1_h_2_*). From the perspective of materials science, a critical temperature zone exists, and it can be changed by the mechanical stress and plastic strain in the machine zone [27]. As shown in Figure 4, the deformation occurs with the cutting temperature decrease from *T_max_* to *T_1_T_2_*. Interval *T_1_*~*T_2_* is considered as the critical cutting temperature zone that results in the phase transformation in cutting operations, and can be changed by the cutting conditions. Then, the machine-affected zone (MAZ) can be estimated by the change of the cutting temperature, namely *h_1_h_2_*, as shown in Figure 4.

**Figure 5:**
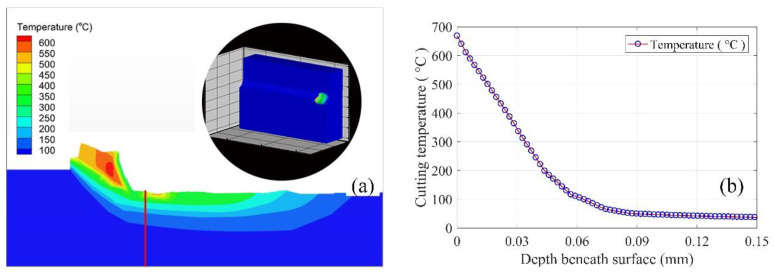
Relationship between cutting temperature and micro-hardness. (**a**) Temperature profile in the cutting zone, (**b**) Temperature beneath the machined surface.

**Figure 6:**
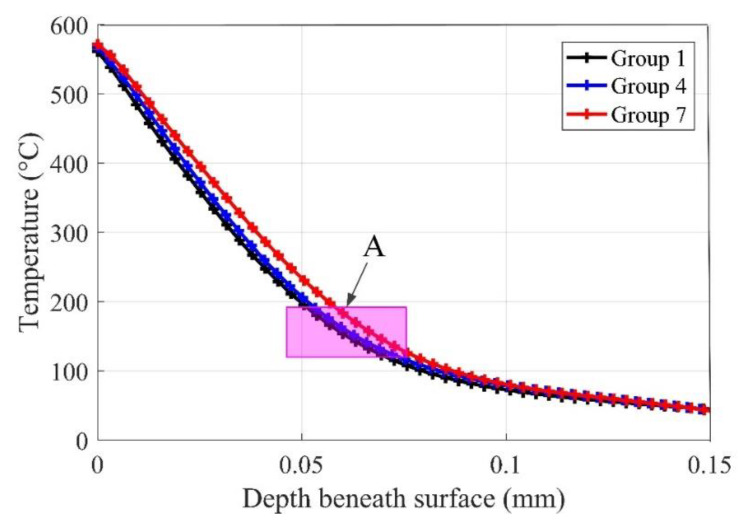
The cutting temperature beneath surface under different cooling methods.

In order to show the distributions of cutting temperature profiles under different coolant methods, finite element method (FEM) simulations are used to find the difference between different methods. There are two constitutive models used in AdvantEdge to model the cutting tests. In this study, standard power law is used to study the performance of cryogenic cooling methods compared with dry machining. In this section, simulations with the parameters given in Table 2 are performed to show the distribution of cutting temperature under the machined surface. Figure 5 shows the sample of temperature distribution simulations and the temperature decrease curves under the surface. Figure 5b is a sample of a decrease of cutting temperature beneath the machined surface obtained from simulation results shown in Figure 5a along the given line. It can be noted that the temperature on the surface is more than 600 °C, but then decreases to normal temperature the along the given line in Figure 5a. Figure 6 shows the temperature decrease along the depth beneath the surface with the cutting speed *v* = 40 m/min and feedrate *f* = 0.06 mm/rev under different cryogenic cooling methods. It can be noted from Figure 6 that the maximum temperature at the surface is approximately equal, while simulations with different cryogenic cooling methods obtain various cutting temperature profiles along the depth beneath the surface. The cutting temperature profile of cooling the cutting zone decreases slower than the others, while the profile of dry cutting decreases faster than precooling the workpiece. This can be explained by the fact that the thermal conductivity of this material decreases with the increasing temperature. Zone A in Figure 6 shows that when the cutting temperature decreases to this zone, the transformation ends, and the microhardness decreases to the hardness of bulk material.

## 3. Results and Discussion

Hardness, one of the most important criteria in material processing applications, has a great effect on tool wear and the fatigue performance of final products. Inconel 718 is susceptible to excessive plastic deformation during the machining processes, due to the high cutting temperature and load. The dislocation generation and movement within the crystal structure of the material, as well as plastic deformation occurring in the cutting process, lead to the strain strengthening of the material [28]. The strain strengthening generated by the plastic deformation in the cutting zone, as well as the high cutting heat, result in the increasing of the surface hardness. In this section, different evaluation criteria are used to give a detailed analysis of the work hardening phenomenon when turning Inconel 718 with a cryogenic assisted machining operation.

### 3.1. Depth of Work Hardening

**Figure 7:**
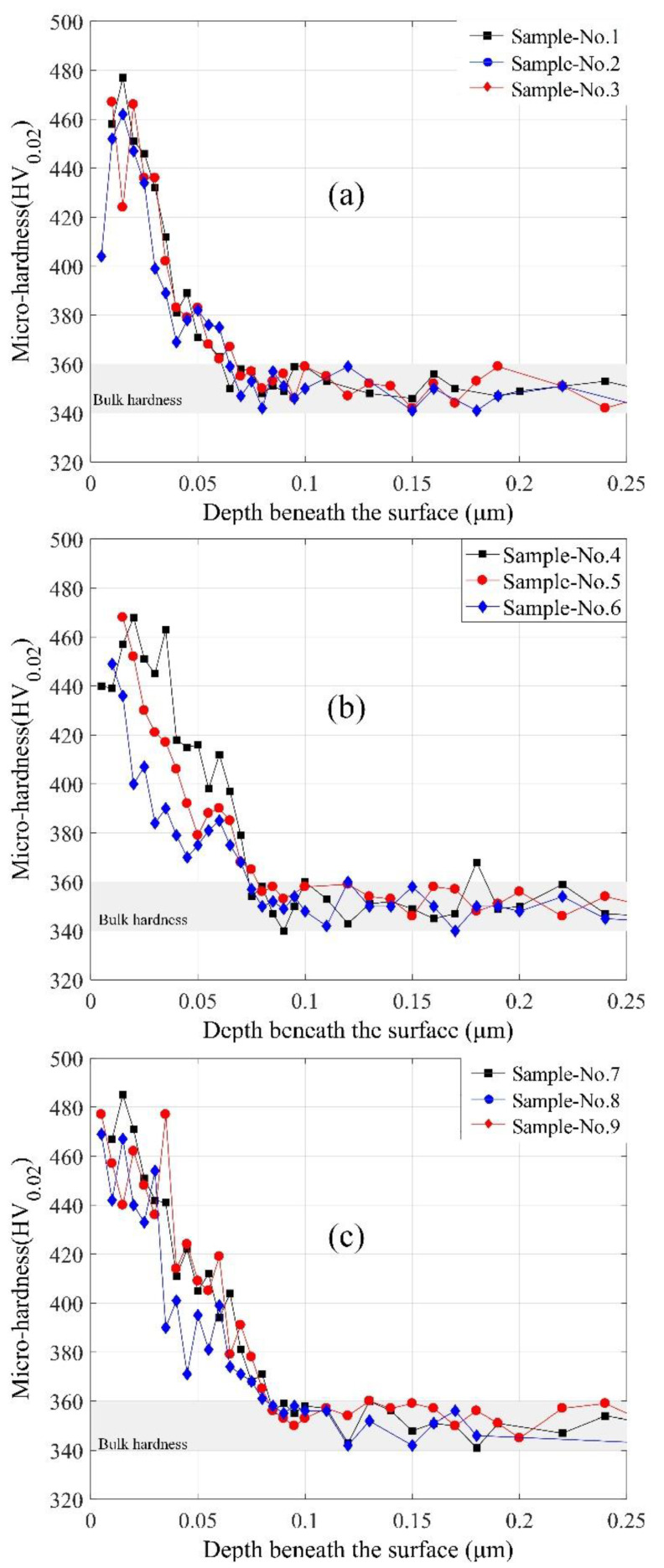
The microhardness beneath machined surface with different cutting conditions: (**a**) dry cutting; (**b**) pre-cooling workpiece; and (**c**) cooling cutting zone.

The microhardness profiles obtained from the samples machined with different cooling conditions are given in Figure 7. It can be noted that the surface hardness near the surface is greater than that of the bulk material. The depth of work hardened layer, which is also called MAZ, is established by the microhardness curve beneath the surface with the hardness of the workpiece. It is evident from Figure 7 that the hardness gradient beneath the surface exists in the region of about 70 μm depth from the surface under different cooling methods. From the test results, it can be noted that the microhardness of the machined surface is larger than the bulk material regardless of the cooling conditions.

It can be noted from Figure 7 that the hardness decreases gradually with the increasing depth beneath the surface. In this paper, the measured profiles with the bulk hardness of Inconel 718, given as about 350 ± 10 HV_0.02_, are used to estimate the machine-affected zone (MAZ). Figure 7a–c indicate the effect of cutting speed on the surface work hardening phenomenon with different cooling conditions. It can be seen from the profiles that the depth of work hardening layer has a slight decrease with the increase of the cutting speed under proposed machining conditions. The depth of the work hardening layer maintains at about 60 μm without coolant—a bit thicker under the pre-cooling method—and then, the highest depth obtained in the cooling cutting zone method (μm). This phenomenon may be resulted by the combination of reduced thermal softening effect and great grain refinement. The cryogenic assisted machining produces higher surface hardness in the machined surface, while dry machining tends to create softer and rougher surfaces due to the lack of coolant. The similar results are found in the turning of Ti6Al4V [29] and burnishing of Co-Cr-Mo biomaterial [22]. The results provide a good agreement to the cutting temperature simulation results shown in Figure 6. For the current work, the correlation between cutting temperature distribution and microhardness depth might lend support to the theory that cutting heat has a significant influence on the change of hardness beneath a workpiece surface.

### 3.2. Yield Strength

**Figure 8:**
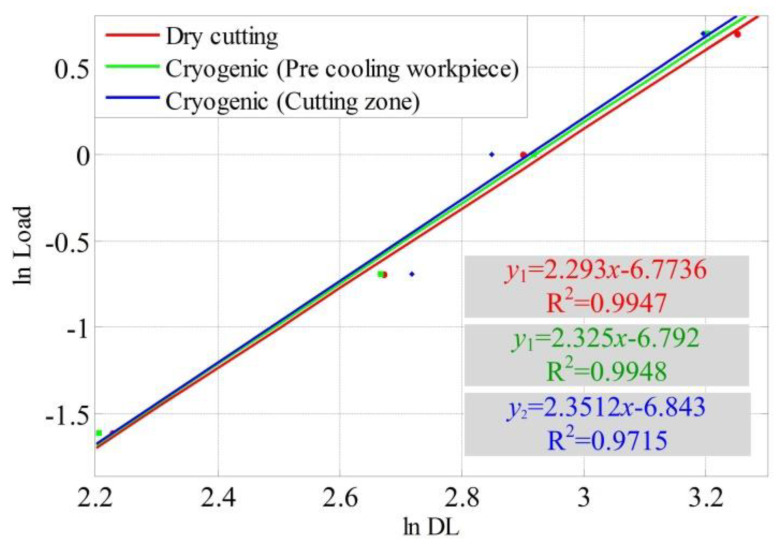
Sample plots showing variation of load with diagonal length. (Sample 1, Sample 5 and Sample 8).

Yield strength is defined as the critical pressure of material property at which plastic deformation takes place. The yield point indicates the upper limit to forces, which can be applied without permanent deformation, and it is a soft failure mode, meaning catastrophic failure or ultimate failure does not occur. It is widely known that the machined surface suffered severe strain hardening during the machining processes, which affects its yield strength. However, it is hard to measure the yield strength in micro sizes with a tensile tester. In this paper, the microhardness beneath the surface is measured and Meyer’s law is used to forecast the yield strength beneath the surface for an in-depth understanding of the deformation of the machined surface. Meyer’s index ‘*m*’ reflects the extent of strain hardening of work hardening in the metal cutting process, and influences the microhardness as well as the yield strength of the material [6]. The ranging test loads from 20 g to 200 g are used to measure the microhardness at a depth of about 30 μm beneath the machined surface, with the samples under different cooling conditions. Then, the average diagonal lengths (ADL) with the fitting data of Equation (1) are presented in Table 3. Meyer’s law illustrates the relationship of load and indentation diagonal length as

(1) $$p=ad^{m}$$

where *p* is the applied load, *d* is the diagonal length of the indentation, *a* is the material constant of workpiece and *m* is the Meyer’s index [30]. Figure 8 shows samples of the plots of load versus ADL with the polynomial fitting curves under different cooling conditions.

(2) Ln(p)=mLn(d)+Ln(a)

From Equation (2), the slope of the straight line can be calculated as *m* and the sample of calculation is shown in Figure 8. Table 3 lists the Meyer’s index acquired from the samples under different cooling conditions. The values of Meyer’s index ‘*m*’ are found to range from 2.293 to 2.326 with dry machining, while it ranges from 2.325 to 2.406 with cryogenic assisted machining conditions. The lower values of the same cutting conditions are used for the evaluation of ultimate tensile strength beneath the machined surface.

Cahoon et al. [31] illustrated the relationship between workpiece hardness and ultimate strength, as shown in Equation (3):

(3) $$\sigma_{u}=\left(\frac{HV}{2.9}\right)\left[1-(m-2)\right]{\left[\frac{12.5(m-2)}{1-(m-2)}\right]}^{\left(m-2\right)}$$

Then, Equation (4) is often used to estimate the yield strength of the material:

(4) $$\sigma_{y}=C_{0}\sigma_{u}$$

*C_0_* in Eqaution (4) is found to be 0.801 [32]. Therefore, Equation (4) becomes:

(5) $$\sigma_{y}=0.801\sigma_{u}$$

By combining Equation (5) with Equation (3), the following Equation (6) can be obtained:

(6) $$\sigma_{y}=0.801\left(\frac{HV}{2.9}\right)\left[1-(m-2)\right]{\left[\frac{12.5(m-2)}{1-(m-2)}\right]}^{\left(m-2\right)}$$

For dry cutting, submitting *n* = *m* − 2 = 0.293 into Equation (6), Equation (7) can be obtained:

(7) $$\sigma_{y}=0.3161HV$$

For the liquid nitrogen coolant pre-cooling workpiece, submitting *n* = *m* − 2 = 0.325 into Equation (6), it will become Equation (8):

(8) $$\sigma_{y}=0.3342HV$$

For the liquid nitrogen coolant, the cutting zone, submitting *n* = *m* − 2 = 0.351 into Equation (6), it will become Equation (9):

(9) $$\sigma_{y}=0.3507HV$$

**Figure 9:**
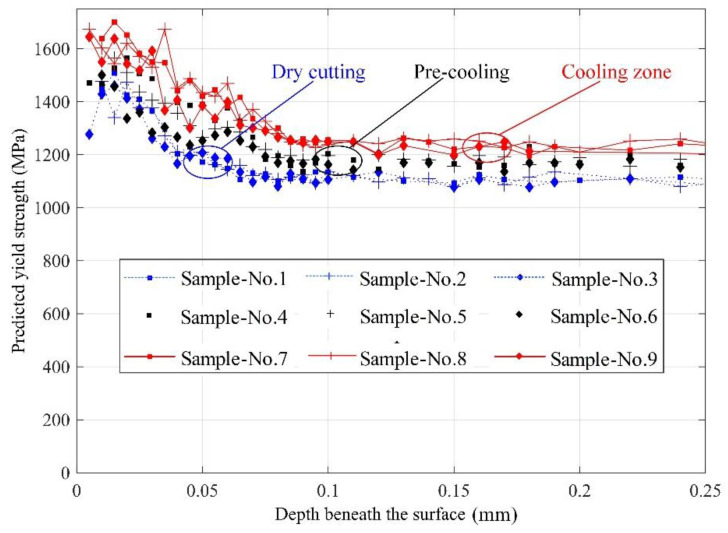
Yield strength with depth below the machined surface under different coolant conditions.

The yield strength at about 30 μm depth beneath the machined surface is calculated by substituting the average microhardness into Equations (7)–(9) with different cooling conditions. Figure 9 shows the yield strength beneath a machined surface with three different cooling conditions. The predicted ultimate yield strength is higher than that of the bulk material regardless of the cooling method. The surface yield strength is found to be about 1700 MPa with cooling the machining zone (Samples 7–9), while it maintains 1500–1600 MPa with precooling the workpiece (Samples 4–6). The cryogenic assisted machining method enhances the yield strength of the surface layer against the dry machining method.

### 3.3. Degree of Work Hardening

As given in Equation (10), the work hardening behavior during machining often defined as the degree of work hardening (DWH)

(10) $$DWH(\%)=\frac{HV_{s}-HV_{b}}{HV_{b}}\times 100$$

where HV_s_ is the surface microhardness and HV_b_ is the bulk material hardness.

The DWH at depths of 30 μm and 50 μm are shown in Table 4, which indicates that the DWH increased with the assisted cooling technology. The microhardness used to calculate the DWH as given in Table 4 are estimated by the exponential fit equation of the measured microhardness. The increasing cutting speed results in the decrease of the DWH under the three types of cooling conditions. It is obvious that the DWH decreases rapidly with the increasing depth, with the DWH given under different measured depths (30 μm and 50 μm).

The hardness increases by 26.77% from 350 ± 10 HV_0.02_ in the bulk material to about 444 HV_0.02_ at about 30 μm below the machined surface without coolant, while it increases by 33.82% with the cryogenic cooling the cutting area. The DWH during pre-cooling of workpiece is slightly smaller than cooling the cutting area. The largest change of microhardness on the machined subsurface obtained by cryogenic machining method is shown in Figure 4, which agrees with the amount of DWH as shown in Table 4. The DWH is slightly reduced with the increased cutting speed when machining with the same cooling conditions, which is consistent with trends of the work hardening curves in Figure 4. Overall, the high DWH and high microhardness can be obtained in the machined surface when machining with a low cutting speed under cryogenic cooling the cutting zone.

## 4. Conclusions

In this work, experiments are employed to study the effect of cryogenic cooling conditions on the generation of a surface hardening layer during turning Inconel 718. It can be concluded the turning processes with the cryogenic cooling method can obtain a higher hardness on the surface layer, which can improve the wear performance of final production compared with dry machining. The main conclusions from the turning experiments can be shown as:

1. The microhardness on the machined surface is significantly higher than the bulk material for the deformation that occurs during cutting processes. The cryogenic assisted technology used in turning processes enhances the parts’ surface integrity in turning Inconel 718. Higher surface hardness can be obtained by cooling the cutting zone rather than precooling the bulk material. In contrast, dry machining trends to generate softer and rougher surfaces for the lack of coolant than assisted machining technologies.
2. The cooling condition has significant influence on the machine-affected zone (MAZ) and degree of work hardening (DWH). The depth of work hardening layer maintains at about 60 μm without coolant, about 70 μm with the pre cooling by liquid nitrogen while reaches about 80 μm with the liquid nitrogen cooling the cutting zone.
3. The surface yield strength of workpiece is predicted by Meyer’s law with the measured microhardness, as well as the indentation diagonal length. The yield strength of the surface with liquid nitrogen cooling is larger than that of dry cutting from this investigation, which enhanced the function performance of the components.

From the view of sustainable manufacturing, cryogenic assisted machining is friendly to the environment, which also provides the parts with higher microhardness and service performance.

## Figures and Tables

**Table 1:** Chemical compositions of the workpiece (Wt.%).

C	Mn	Si	P	Ni	Cr	Mo	Ti	Nb	Co	B	Al	Fe
0.03	0.02	0.09	0.003	52.48	18.94	3.03	0.98	5.13	0.02	0.003	0.51	other

**Table 2:** Experimental cutting parameters (*f* = 0.06 mm/rev).

No.	Cooling Method	Cutting Speed (m/min)
1	Dry	40
2		60
3		80
4	Cryogenic (pre-cooling workpiece)	40
5		60
6		80
7	Cryogenic (cooling cutting zone)	40
8		60
9		80

**Table 3:** Indentation load with the corresponding diagonal length from the samples about 30 μm deep from the machined surface.

Sample	Diagonal Length at Different Loads (μm)				m	R^2^
	20 g	50 g	100 g	200 g		
1	9.28	14.47	18.17	25.76	2.293	0.9947
2	8.95	14.57	18.31	24.18	2.326	0.9895
3	9.12	14.78	17.99	25.11	2.310	0.9886
4	9.43	13.46	16.98	25.59	2.332	0.9854
5	9.08	14.39	18.51	24.55	2.325	0.9948
6	9.01	14.37	17.29	24.19	2.374	0.9879
7	8.96	14.18	16.98	23.77	2.405	0.9876
8	9.02	15.13	17.25	24.38	2.351	0.9715
9	8.89	14.79	17.93	23.09	2.406	0.9775

**Table 4:** The DWH at different depths beneath the machined surface.

Sample	Depth	1	2	3	4	5	6	7	8	9
DWH (%)	30 μm	26.77	22.77	24.25	31.41	28.46	19.59	33.82	27.62	25.72
DWH (%)	50 μm	8.51	6.76	6.79	15.22	10.30	6.3	15.26	14.90	10.91
